# Antibiotic practices in kidney transplant recipients with urosepsis are associated with treatment outcomes – a post-hoc analysis of an observational study

**DOI:** 10.1007/s10096-025-05181-x

**Published:** 2025-06-09

**Authors:** Tomasz Królicki, Tobiasz Kudla, Anna Królicka, Klaudia Bardowska, Krzysztof Letachowicz, Ryszard Gawda, Tomasz Czarnik, Magdalena Krajewska, Dorota Kamińska

**Affiliations:** 1 Present Address: Department of Anesthesiology and Intensive Care, Institute of Medical Sciences, University of Opole, Opole, Poland; 2 Department of Nephrology, Transplantation Medicine and Internal Diseases, Wrocław Medical University, Opole, Poland; 3 Department of Non-Procedural Clinical Sciences, Faculty of Medicine, Wroclaw University of Science and Technology, Opole, Poland

**Keywords:** Urosepsis, Urinary tract infection, Empiric antibiotic therapy, Sepsis, Kidney transplantation

## Abstract

This study is a post-hoc analysis of a previously published observational study. The aim is to characterize the epidemiology, etiology and antibiotic practices in KTRs admitted due to urosepsis (US) and urinary tract infection (UTI) to a tertiary transplantation center between the period 2014–2019.The two study groups consisted of 101 KTRs with a primary episode of US and 100 randomly selected KTRs hospitalized for UTI as a control group. A post-hoc analysis of clinical and microbiological data was performed, and a local microbiological map was created.The estimated yearly incidence of US in the study population was 21.1 (95% CI: 15.3–26.9) cases per 1000 KTRs per year. Urosepsis accounted for 85% of all sepsis cases. The most common causative pathogen in both groups was *Escherichia coli*. In 46% and 11% of US and UTI cases, initial empiric antibiotic therapy had to be escalated until day 3 due to lack of clinical response or proven bacterial resistance. This delay in appropriate antibiotic treatment in the US group was significantly associated with mortality (OR = 10.1 with *p* = 0.021), need for acute renal replacement therapy (OR = 4.73 with *p* = 0.012) and non-recovery from AKI (OR = 3.18 with *p* = 0.031). After discharge from hospital, almost 60% of all study subjects received a prophylactic antibiotic course for prevention of UTI recurrence, which did not translate into a lower frequency of readmission due to UTI in a multivariable analysis.US is a common insult that contributes to graft failure in KTRs. We recommend adjusting US and UTI treatments to center-specific microbiological maps in order to optimize treatment outcomes, reduce development of antibiotic resistance, and avoid unnecessary exposure to nephrotoxic antimicrobial drugs.

**Supplementary Information:**

The online version contains supplementary material available at 10.1007/s10096-025-05181-x.

## Introduction

Kidney transplant recipients (KTRs) are exceptionally susceptible to both cardiovascular disease and infections. The majority of recorded infections in this patient group are comprised of urinary tract infections (UTIs) [1]. A recent meta-analysis of epidemiological studies showed that the prevalence of urosepsis (US) among KTRs was 35% [2]. As cardiovascular events are the main source of mortality in KTRs, UTIs are a significant contributor to graft failure and its subsequent loss [3]. Chronic immunosuppression, frequent hospitalizations, exposure to dialysis and altered urinary tract anatomy with residual foreign bodies (such as ureteral stents) seem to be the most important contributors that predispose KTRs to the development of UTIs and frequent development of urosepsis (US) [4–6].

Although the treatment of US and UTIs has been standardized by the European Association of Urology (EAU) and Surviving Sepsis Campaign (SSC) guidelines, the population of KTRs requires a personalized approach, due to the specific risk factor profile and high exposure to healthcare facilities and hospital microbiota [7–9].

The urinary pathogens causing complicated UTIs in the general population are characterized by high antibiotic resistance rates, also against broad-spectrum agents [10]. The data on antibiotic resistance rates in KTRs with UTIs or US are limited. Furthermore, KTRs have an exceptionally high predisposition for urinary tract colonization and UTI recurrence. In fact, UTI may affect up to 72% of kidney recipients [11]. Although guidelines do not support the routine use of prophylactic antibiotic therapy in KTRs to prevent recurrence of UTIs, the scientific literature on this topic is conflicting [9, 12].

The primary aim of this study was to characterize the epidemiology and etiology of US and UTI, as well as to describe antibiotic practices in a population of KTRs in a tertiary transplantation center in Wroclaw, Poland from 2014 to 2019 [13].

## Materials and methods

### Study design

The study was designed as a post-hoc analysis of a previously published retrospective cohort study [13]. It was performed on the whole population of kidney transplant recipients who are under the care of the Transplantation Clinic of Wroclaw Medical University. Approval of the local bioethical committee was obtained in advance of the start of the study (approval number KB-775/2018). The study was performed in agreement with the Declaration of Helsinki.

### Study objectives

The aim of the study was to assess the incidence of urosepsis and urinary tract infections in the population of KTRs. Further objectives included an evaluation of (i) the etiology of US and UTI, (ii) the frequency of multidrug-resistant bacterial strains, and (iii) local antibiotic practices. A local antibiotic resistance map was created, and antibiotic practices were correlated with patient- and kidney graft related outcomes.

### Data

All records of KTR hospitalizations for 2014–2019 were screened (using ICD-10 codes) for urosepsis, urinary tract infections and other infections.

Incidence of US and UTI was estimated based on the total number of cases and an assumption of the KTR population size, which was set for a fixed number of 1100 KTRs. Precise numbers could not be provided due to dynamic changes that occur in this population (constantly occurring deaths or graft loss with return to dialysis and new transplantations). The minimal and maximal KTR count registered in the outpatient Transplantation Clinic during the study period ranged from 1043 to 1121.

To provide data on the etiology of investigated infections, all first-time cases of urosepsis in KTRs were included. As a control group, 100 cases of KTRs hospitalized for UTI were included as described in the primary study [13]. Appropriate clinical, laboratory and microbiological data were harvested from patients’ documentation. All patients were followed for one year using data from the Outpatient Transplantation Clinic of the Wroclaw Medical University.

Clinical data from our previous study (DOI: 10.17632/m323c88sy4.2) were used to investigate any possible link between antibiotic practices and treatment outcomes. All definitions and criteria used in the study are summarized in the Supplementary Information in Sect. 1.1.

### Microbiologic data

All culture (urine, blood and other) results from the hospitalized patients were obtained. The pathogens were classified as multi-drug-resistant (MDR) when they were resistant to ≥ 1 or more classes of antimicrobial agents - according to the joint definition provided by the European Centre for Disease Prevention and Control (ECDC) and Centers for Disease Control and Prevention (CDC) [14]. The susceptibility profiles were identified by the microbiologic laboratory using the European Committee on Antimicrobial Susceptibility Testing (EUCAST) recommendations [15]. The microbiologic map generated from the obtained microbiologic data was created according to CLSI guidelines and the methods are described in detail in Section S1.3. of the Supplementary Information [16, 17].

### Antibiotic practices

Each patient’s course of treatment was evaluated to determine the type of empiric antibiotic used. Patients with dual therapy were presented separately. Inappropriate empiric antibiotic therapy was defined as the need to escalate antibiotic treatment up to 72 h after diagnosis, due to either proven antimicrobial resistance in urine or blood cultures, or clinical deterioration (mainly persisting or aggravating symptoms or worsening graft or native organ function). Empiric treatment success rate was calculated based on this definition.

### Statistical analysis

Binominal and discrete data were presented as total counts and percentages. Estimates of incidence were presented as counts (per 1000 KTRs / per study year) with 95% confidence intervals (95% CI). The Shapiro–Wilk test was used for continuous variables to assess the assumption of normality, which were presented as either mean and standard deviation (SD) or median and interquartile range (IQR), as appropriate. The significance of differences between two groups was tested using an independent Student t-test for normally distributed variables and the Mann-Whitney U-test for skewed variables. To assess the risk of certain complications in exposed and unexposed patients, odds ratios (OR) with 95% CIs were calculated. The significance was tested using the Fisher exact test.

To investigate risk factors of UTI recurrence, a multivariable Cox hazards regression model with stepwise elimination of variables was used. Additionally, variables which were deemed to be of clinical significance, irrespectively of their p-value, were added to the model. The proportional hazards assumption was tested graphically using an analysis of Kaplan Meier curves and Schoenfeld plots, and tested using a PH test. A two-tailed p-value of < 0.05 was set as statistically significant. All analyses were conducted using Statistica v. 13.2 (StatSoft Inc., Tulsa, Oklahoma, US).

## Results

### Incidence of US and UTI as bacterial infections in KTRs

**Fig. 1:**
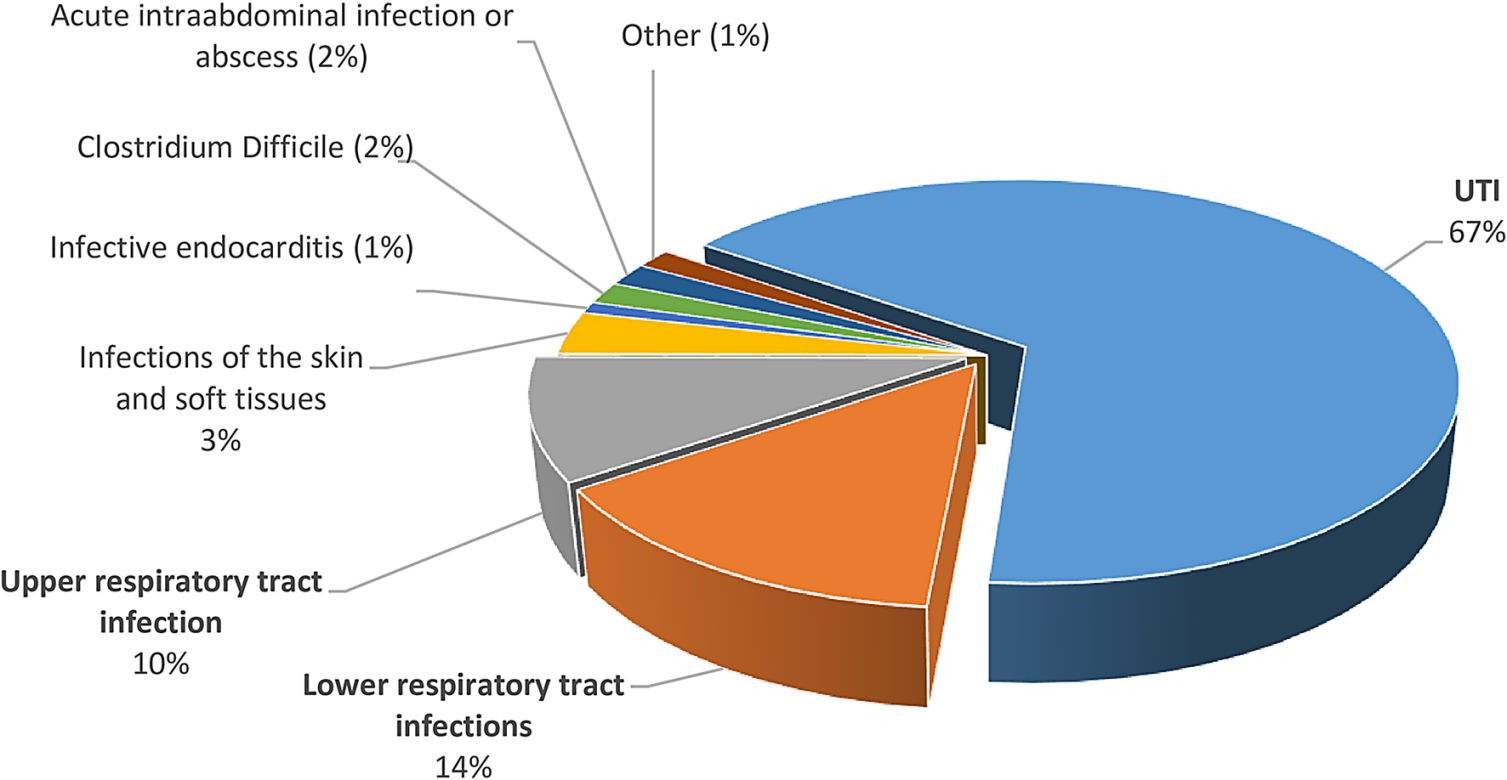
Proportions of all registered infection types diagnosed in the study cohort between 2014 and 2019

From 2014 to 2019, we identified 139 KTRs hospitalized for urosepsis, which accounted for 85% of all sepsis cases in this patient group. The total number of UTI cases (including US) registered in the study was 715, which contributed to almost 10% of all hospitalizations in our Transplantation Clinic. The cases of UTI accounted for approximately two-thirds of all registered bacterial infections – as presented in Fig. 1.

The bare counts of US, sepsis (all causes), UTI and infections, as well as percentages of all hospitalizations for each infection type (per study year), are described in Supplementary Table S1. The estimated yearly incidence of US was 21.1 (95%CI: 15.3–26.9) cases / 1000 KTRs / year – as presented in Table S2. Additional data, distinguishing between infection types, are shown in an additional Table in Section S3 of the supplementary materials.

### US and UTI – group characteristics

In the total of 139 US cases, only patients with primary US episodes were included. Therefore only 101 cases of urosepsis were analyzed, which comprise the US group. Of the 715 UTI cases, 100 patients were randomly chosen as a control group (UTI - group). The baseline characteristics of the US- and UTI-group, as well as their treatment outcomes, are presented in Tables S4 and S5, Section S2. No differences regarding basic data and baseline allograft function were found.

The Gram (-) strains accounted for 78% and 59% of all US and UTI cases, respectively. The most common strain causing both US and UTI was *Escherichia coli*, followed by *Klebsiella Pneumoniae* and *Enterococcus faecalis*. The etiology was unspecified in 24% and 12% of the US and UTI cases, respectively. Table 1 summarizes the etiology of both US and UTI cases, including the rates of MDR (multidrug-resistant) strains. The most prevalent MDR mechanism was ESBL (Extended Spectrum Beta Lactamase) in Gram-negative bacteria, which was up to 70% in cases infected with *Klebsiella Pneumoniae*.

**Table 1:** Etiology of Urosepsis and urinary tract infection with rates of multi-drug resistant bacterial strains

	US (*n* = 101)	MDR mechanism and its frequency in each of the pathogen group	UTI (*n* = 100)	MDR mechanism and its frequency in each of the pathogen group	*p*-value*
*Escherichia coli* [n, %]	45 (44.5%)	ESBL (13.3%)	32 (32%)	ESBL (6.25%)	0.5320
*Klebsiella pneumoniae* [n, %]	21 (20.8%)	ESBL (66.7%)	17 (17%)	ESBL (70.6%)	0.9999
*Enterococcus faecalis* [n, %]	5 (4.9%)	HLAR (20%)	13 (13%)	HLAR (15.4%)	0.6379
*Proteus mirabilis* [n, %]	4 (4%)	-	2 (2%)	-	n/a
*Staphylococcus aureus* [n, %]	3 (3%)	MRSA (33.3%)	0 (0%)	-	n/a
*Enterococcus faecium* [n, %]	3 (3%)	VRE (66.7%)	2 (2%)	HLAR (50%)	n/a
*Staphylococcus spp*.** [n, %]	3 (3%)	MLSB (33.3%)	0 (0%)	-	n/a
*Enterobacter cloacae* [n, %]	2 (2%)	ESBL (100%)	1 (1%)	ESBL (100%)	n/a
*Pseudomonas aeruginosa* [n, %]	1 (1%)	-	2 (2%)	-	n/a
*Morganella morganii* [n, %]	0 (0%)	-	3 (3%)	-	n/a
*Enterobacter aerogenes* [n, %]	0 (0%)	-	2 (2%)	-	n/a
*Citrobacter freundi* [n, %]	0 (0%)	-	1 (1%)	-	n/a
Mixed etiology	2 (2%)	-	1 (1%)	-	n/a
Unknown*** [n, %]	12 (11.8%)	-	24 (24%)	-	n/a
MDR strains - overall [n, %]	29 (28.7%)	-	19 (19%)	-	0.1363

ESBL – extended spectrum beta-lactamase, HLAR – high level aminoglycoside resistance, MDR – multi-drug-resistance, MLSB - macrolide lincosamide streptogramin B resistance, MRSA – methicillin-resistant Staphylococcus aureus, n/a – not applicable, VRE – vancomycin resistant enterococcus

*p-value for comparison of MDR frequency in each bacteria species

**Staphylococcus epidermidis,* Staphylococcus haemolyticus*,* Staphylococcus hominis.*

*** no reliable blood or urine culture was obtained, evident culture contamination or negative cultures were present

Table 2 presents the frequencies of antibiotic resistance registered in Gram-negative strains in both the US and UTI groups, based on a standard resistance-antibiotic set analysed in our microbiological laboratory. The resistance rates against agents commonly used in ambulatory treatment, such as ciprofloxacin or trimethoprim with sulfamethoxazole (TMP/SMX), accounted for up to 95% of the cultures analyzed, depending on the isolated pathogen type. The overall resistance rates against aminopenicillins and II-/ III-generation cephalosporines ranged between 25 and 30%. However, in the subgroup of patients with US and UTI caused by *Klebsiella pneumoniae*, the resistance rates ranged from 50 to 70% and 40–60%, respectively. Resistance against carbapenems (ertapenem, meropenem and imipenem) was also registered among *Klebsiella* and *Enterobacter* strains, however it did not exceed 3% of all Gram-negative infections. Patients with US presented a similar frequency of MDR strains to their UTI counterparts.

**Table 2:** The prevalence of antibiotic resistances identified in cultures with gram (-) bacterial growth in patients with Urosepsis and urinary tract infection

		Ciprofloxacin	TMP/SMX	Amoxicillin	Cefuroxime	Cefotaxime	Ceftazidime	Piperacillin / Tazobactam	Cefepime	Imipenem	Meropenem	Ertapenem
*Escherichia coli*	Urosepsis (*n* = 48)	15 (31%)	20 (42%)	7 (15%)	4 (8%)	5 (10%)	4 (8%))	5 (10%)	2 (4%)	0 (0%)	0 (0%)	0 (0%)
	UTI (*n* = 29)	16 (55%)	16 (55%)	10 (35%)	5 (17%)	2 (7%)	2 (7%)	2 (7%)	1 (4%)	0 (0%)	0 (0%)	0 (0%)
	Overall (*n* = 77)	31 (40%)	36 (47%)	17 (22%)	9 (12%)	7 (9%)	6 (8%)	7 (9%)	3 (4%)	0 (0%)	0 (0%)	0 (0%)
*Klebsiella pneumoniae*	Urosepsis (*n* = 23)	19 (83%)	20 (87%)	12 (52%)	16 (70%)	16 (70%)	13 (57%)	16 (70%)	11 (48%)	0 (0%)	0 (0%)	1 (4%)
	UTI (*n* = 19)	18 (95%)	18 (95%)	15 (79%)	11 (58%)	12 (63%)	7 (37%)	7 (37%)	7 (37%)	0 (0%)	0 (0%)	2 (11%)
	Overall (*n* = 42)	37 (88%)	38 (91%)	27 (64%)	27 (64%)	28 (67%)	20 (48%)	25 (60%)	18 (43%)	0 (0%)	0 (0%)	3 (7%)
Other Gram (-) bacilli*	Urosepsis (*n* = 8)	6 (75%)	6 (75%)	1 (13%)	1 (13%)	3 (38%)	3 (38%)	2 (25%)	3 (38%)	1 (13%)	0 (0%)	0 (0%)
	UTI (*n* = 8)	2 (25%)	1 (13%)	3 (38%)	1 (13%)	1 (13%)	1 (13%)	4 (50%)	1 (13%)	1 (13%)	1 (13%)	1 (13%)
	Overall (*n* = 16)	8 (50%)	7 (44%)	4 (25%)	2 (13%)	4 (25%)	4 (25%)	6 (38%)	4 (25%)	2 (13%)	1 (6%)	1 (6%)
Overall: Gram (-) pathogens	Urosepsis (*n* = 79)	40 (51%)	46 (58%)	20 (25%)	21 (27%)	24 (30%)	20 (25%)	23 (29%)	16 (20%)	1 (1%)	0 (0%)	1 (1%)
	UTI (*n* = 56)	36 (64%)	35 (63%)	28 (50%)	17 (30%)	15 (27%)	10 (18%)	13 (23%)	9 (16%)	1 (2%)	1 (2%)	3 (5%)
	Overall (*n* = 135)	76 (56%)	81 (60%)	48 (36%)	38 (28%)	39 (29%)	30 (22%)	38 (28%)	25 (19%)	2 (2%)	1 (1%)	4 (3%)

*Acinetobacter baumannii,* Enterobacter aerogenes*, *Enterobacter cloacae*, *Morganella morganii*, *Proteus mirabilis*, *Proteus vulgaris*, *Pseudomonas aeruginosa*

### Antibiotic practices and their association with outcomes

Table 3 summarizes the primary antibiotics used in the study cohort, as well as their effectiveness in empiric therapy. A total of 87% of the US cases were treated primarily with piperacillin with tazobactam, or higher tier antibiotics (third generation cephalosporins, carbapenems) and in 25 subjects, vancomycin was added to a beta-lactam regimen.

**Table 3:** Choice of primary antimicrobial agents in KTRs hospitalized for urosepsis and urinary tract infection

	Urosepsis (*n* = 101)	UTI (*n* = 100)	*p*-value
Time of antibiotic administration (median, IQR) [days]	13 (10–14)	7 (6–10)	< 0.0001
Amoxicillin with clavulanic acid [n, %]*	18 (16.7%)	47 (89.4%)	< 0.0001
Piperacillin with tazobactam [n, %]*	7 (57.1%)	5 (60%)	0.6207
Ciprofloxacin [n, %]*	4 (0%)	8 (62.5%)	0.1473
Cephalosporine II - generation [n, %]*	3 (66.6%)	6 (100%)	0.3333
Cephalosporine III - generation [n, %]*	25 (76%)	13 (92.3%)	0.4300
Carbapenems [n, %]*	15 (100%)	10 (100%)	0.9999
Vancomycin with beta-lactam [n, %]*	23 (26.1%)	2 (100%)	0.1741
Need for antibiotic escalation due to lack of disease control or proven resistance [n, %]	46 (45.5%)	11 (11%)	< 0.0001
Prophylactic antibiotic therapy after discharge [n, %]	58 (57.4%)	64 (64%)	0.3871
Time of antibiotic administration post discharge [days]	10 (7–30)	10 (7–20)	0.5038

*Percentages given in brackets refer to the efficiency of the primary AB-regimen, defined as no need for antibiotic escalation in order to control symptoms and successfully treat underlying infection. P-value in these cases refers to absolute numbers

In the US and UTI groups, marked by the cumulative need for antibiotic escalation after 72 h of treatment, inappropriate empiric therapy had been administered in 45.6% and 11% of cases, respectively. Empiric UTI treatment agents such as amoxicillin with clavulanic acid or ciprofloxacin showed acceptable efficiency, with success rates of 89% and 63%, respectively. However, in the US group, these two antibiotics were shown to be inappropriate (17% and 0% effectiveness, respectively). Piperacillin with tazobactam showed a similar treatment success rate in both groups, reaching up to 60%.

All the higher-tier antimicrobials (second and higher generation cephalosporines and carbapenems) showed a gradually increasing efficiency, reaching 100% for carbapenems. Poor treatment outcomes (26%) were however registered in KTRs with urosepsis who were treated primarily with vancomycin and other beta-lactam antibiotics (various agents from amoxicillin to carbapenems).

We also analyzed the potential association between inappropriate antibiotic treatment and patient outcomes. In the US group, an inappropriate selection of antimicrobial agent was correlated with increased risk of death (OR = 10.1 with *p* = 0.021), need for acute renal replacement therapy (OR = 4.73 with *p* = 0.012) and non-recovery from AKI (OR = 3.18 with *p* = 0.031). A similar, but not statistically significant trend was observed in the UTI group for non-recovery from AKI (OR = 1.64 with *p* = 0.636). In both groups the length of hospital stay was significantly higher in patients who did not receive the appropriate empiric antimicrobial treatment.

### Antibiotic prophylaxis of relapse and recurrence of UTI after discharge

In both US and UTI groups, in approximately 60% of cases a prophylactic antibiotic treatment was prescribed after discharge. Three agents: amoxicillin with clavulanic acid, TMP/SMX and ciprofloxacin were used for post-discharge antibiotic therapy. Relapse of UTI occurred almost exclusively in the US group. In patients with urosepsis, antimicrobial prophylaxis post discharge was not associated with UTI relapse (OR = 0.68; 95% CI: 0.22–2.06, *p* = 0.4913).

**Fig. 2:**
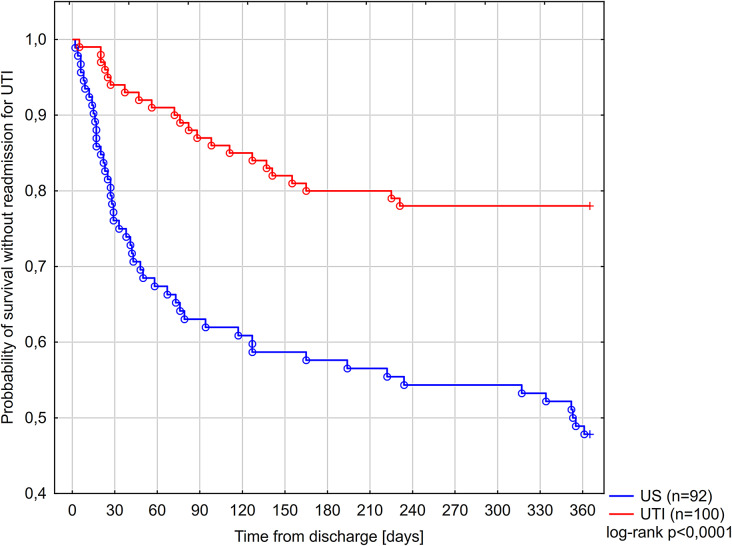
Kaplan Meier curve for readmission due to UTI in both study groups during one year follow-up

Figure 2 presents a Kaplan-Meier curve for readmission due to UTI in both study groups during the one year follow-up. Four key factors that were independently associated with re-hospitalization for UTI within one year were: (i) the development of urosepsis, (ii) urine outflow obstruction, (iii) UTI caused by MDR strain, and (iv) prior history of recurrent UTI. Prophylactic antibiotic therapy after discharge appeared to have no significant effect on UTI rehospitalization rate. The model used for predictor assessment is presented in Section S6.

## Discussion

Our study confirms previous reports that the incidence of sepsis in the population of KTRs is substantially higher than in the general population [18]. Furthermore, UTI was the dominant type of infection among KTRs admitted to the hospital and accounted for 67% of all infection cases in our study group. Although the mortality related to urosepsis among KTRs was relatively low and did not exceed 10%, it was still twice as high as in the general population [19]. Furthermore, we found that US poses a common insult to kidney graft and contributes significantly to its failure and subsequent loss. A microbiological map of KTRs admitted to our center due to US and UTI revealed that the bacterial pathogens responsible for infection resemble a highly resistant hospital microbiome – which is in line with observations from the general patient population [10].

The collected data also enabled us to characterize local antibiotic practices. First, the median time of antibiotic therapy in patients with US (median of 13 days) was substantially longer than the mean antibiotic course that is recommended by EAU (7–10 days) and SSC guidelines in the general population (5–10 days with recommendation towards shorter courses) [7, 8]. Conversely, the guidelines of the American Society of Transplantation recommend even longer 14–21-day courses of targeted narrow spectrum antibiotic therapy for complicated pyelonephritis or urosepsis [9].

We believe that such long courses seem excessive, especially in the light of emerging evidence that in the case of adequate source control, antibiotic treatment can be safely discontinued at day 4 to 5 [20, 21]. Although in our study, an appropriate choice of antimicrobial agent for the treatment of US (as per EAU guidelines) was observed in the majority of study cases, the frequency of inappropriate empiric treatment was 46% [8]. This partially explains the longer median times of antibiotic treatment, which even with this prolongation, can be considered as “longer antibiotic courses” as per SSC nomenclature [7].

The main contributory organisms identified in our cohort were similar to those found in the general population, with *Escherichia coli* being the most common microorganism, followed by *Klebsiella pneumoniae* and other Gram-negative pathogens, irrespectively of septic status. In addition, the frequency of MDR was not associated with infection phenotype (US vs. UTI), but rather with the microorganism species, with the most common being among *Klebsiella pneumoniae* isolates.

Our study also indicates that in settings where ciprofloxacin and TMP/SMX were used very frequently (for UTI and *Pneumocystis jiroveci* prophylaxis in the early post-transplant period), very high resistance rates were observed which reached 85–90% of all cultures (Table 2). Therefore, in the settings described these two antibiotics along with amoxicillin should not be used for the treatment of US or complicated pyelonephritis, as indicated by EAU and AST [8, 9]. The recent update of EAU guidelines recommends against the use of ciprofloxacin in any patient with pyelonephritis who is being treated in as an inpatient, has a history of previous ciprofloxacin use within the previous 6 months and when anticipated resistance rates of the causative pathogens exceed 10% [22]. On the other hand, IDSA guidelines regarding uncomplicated pyelonephritis refrain from defining the threshold of local ciprofloxacin resistance, which should trigger the use of higher tier antibiotics [23]. It therefore seems that ciprofloxacin and TMP/SMX should not be used for the empiric treatment of any form of UTI in kidney transplant patients. However, in cases with proven microbial susceptibility, they could be used as targeted therapy.

Our results also clearly highlight the association between a delay in appropriate antibiotic therapy and detrimental patient and kidney graft outcomes. The need for antibiotic escalation due to empiric agent failure was associated with an increased risk of death, the need for renal replacement therapy and non-recovery from AKI at one month. Recent insights from the SERPENS study, as well as other smaller reports, confirm the association between inappropriate empiric antibiotic therapy and mortality in US in the general population, despite a generally favorable prognosis with low mortality risk compared to other sources of infection [19, 24]. To date, we are not aware of any reports that analyze the outcomes of US in KTRs, in terms of antibiotic practices.

The presented association between graft function and inappropriate antibiotic selection seems to be in line with common knowledge. Therefore, in cases of evident US or severe UTI, physicians should choose antibiotics with proven activity against local microflora (in our population, third generation cephalosporins, piperacillin with tazobactam or carbapenems). In patients presenting with shock or rapidly deteriorating, the empiric use of carbapenems seems to be justified as it presented almost 100% activity in the harvested Gram-negative cultures. It is also worth mentioning that in only one patient in the US group, did the microbiologic cultures justify the use of vancomycin (with methicillin-resistant *Staphylococcus aureus*). The microbiologic map showed that the absolute majority of microorganisms (both Gram-positive and negative) were susceptible to some form of beta-lactams and other non-glycopeptide antibiotics. We therefore suggest that dual empiric vancomycin and beta-lactam therapy should only be used in KTRs presenting with US and septic shock. Excessive and unjustified use of vancomycin may promote the development of antimicrobial resistance, increase the rate of *Clostridioides difficile* infections and lead to additional kidney graft injury [25]. A growing body of evidence indicates that the concomitant use of vancomycin and piperacillin with tazobactam may further increase the risk of acute kidney injury [26, 27].

Finally, we also analyzed the frequency of prophylactic antibiotic therapy, which was prescribed to KTRs after hospital discharge, after a US or UTI episode. Approximately 60% of our study population received a prophylactic antibiotic course, both in subjects within and after the first 6 months post-transplantation. Its use did not reduce UTI recurrence, either due to high resistance rates against the antibiotics prescribed or due to the futility of such an intervention. In a multivariable analysis, the only modifiable risk factor contributing to the recurrence of UTI was the presence of urine outflow obstruction (both at the lower- and upper urinary tract level). In selected cases, the obstruction was treated by bladder catheterization or ureteral stent implantation. It is worth mentioning that our model presented in Table S6 reached a low R^2^ of 0.4851, which indicates that there is still a large portion of variance that needs to be explained by other measurable or non-measurable factors. A meta-analysis conducted by Green et al., showed a neutral effect of antibiotic UTI-prophylaxis in the first 6 months after kidney transplantation on mortality and graft loss [12]. The authors also reported that antibiotic prophylaxis reduced the incidence of urosepsis and bacteriuria but significantly increased the proportion of UTIs with pathogens that were identified as TMP/SMX resistant. We hypothesize that in our study, the lack of prophylaxis efficiency was associated with widespread resistance against the most commonly used antibiotics. Therefore, as AST guidelines indicate, antibiotic prophylaxis of UTI should only be introduced in highly selected cases and should be based on actual urine culture with antibiogram [9].

Based on the experience from our center as well as analyses of the literature, we propose that in every kidney transplantation ward, a local microbiological resistance map should be created and regularly updated to guide rational antibiotic therapy, reduce the development of antimicrobial resistance, and minimize unnecessary exposure to potentially nephrotoxic drugs. The use of rapid multiplex polymerase chain reaction-based (PCR) diagnostic testing may also improve antibiotic use and reduce the time to commencement of appropriate antimicrobial therapy [28, 29]. In the absence of guidelines for the treatment of US and sepsis in KTRs, there is a significant need for randomized clinical trials in order to develop strategies to preserve graft function, especially when there is a donor shortage.

### Limitations and strengths

The main limitation of our study is its retrospective nature and relatively small sample size of patients recruited. Although the low case volume of US did not enable us to create a microbiological map with an optimal single isolate sample size of *n* = 100 (as indicated by CLSI), a minimal sample size of 30 isolates for *Escherichia coli* and *Klebsiella pneumoniae* was achieved [16, 17]. Another drawback is the lack of reliable data on antibiotic dosage. We were therefore unable to comment on the efficiency of maximal dosing or GFR-adjusted dosing strategies or continuous beta-lactam infusion regimens in the study setting. Despite these limitations, our study highlights the importance of creating microbiological maps and antibiotic stewardship, even in such small populations as kidney transplant recipients.

## Conclusions

Urosepsis is the most prevalent type of sepsis in KTRs, which has a substantial negative impact on graft function. Our study shows a clear association between delay in effective antimicrobial treatment in urosepsis in KTRs and detrimental kidney graft outcomes. We suggest that the creation of local antimicrobial resistance maps may aid clinicians in adjusting antibiotic treatment to individual patient needs. Routine antimicrobial prophylaxis in KTRs after urinary tract infection, without considering the local antibiotic resistance profile, may render it futile and lead to antibiotic resistance. Unsupervised antibiotic therapy may also lead to inadvertent exposure of KTRs to nephrotoxic drugs.

## Electronic supplementary material

Below is the link to the electronic supplementary material.

Supplementary Material 1

## Data Availability

The dataset that was analysed during the current study is available in the Mendeley repository under the link: https://data.mendeley.com/drafts/m323c88sy4. The study dataset that has been published in repository has been ridden of information that would allow potential re-identification of study subjects.

## Declarations

### Affiliations

Most of the study authors have a dual affiliation due to subsequent change of place of work during the study or shortly after its completion.

## References

[1] Alangaden GJ, Thyagarajan R, Gruber SA, Morawski K, Garnick J, El-Amm JM et al (2006) Infectious complications after kidney transplantation: current epidemiology and associated risk factors. Clin Transpl 20:401–409. 10.1111/j.1399-0012.2006.00519.x 10.1111/j.1399-0012.2006.00519.x 16842513

[2] Hosseinpour M, Pezeshgi A, Mahdiabadi MZ, Sabzghabaei F, Hajishah H, Mahdavynia S (2023) 1;24 Prevalence and risk factors of urinary tract infection in kidney recipients: a meta-analysis study. BMC Nephrol 10.1186/s12882-023-03338-4 10.1186/s12882-023-03338-4 PMC10523791 37759155

[3] Hamilton ADM, Prætorius HA (2024) Reduced graft survival in renal transplant patients with urinary tract infections – a meta-analysis. Dan Med J 71. 10.61409/A06230424 10.61409/A06230424 38314732

[4] Omic H, Eder M Effect of increasing age and ureteral stent implantation on urinary tract infections after kidney transplantation - update of recent literature. Curr Opin Urol 2024 1;34:146–153. 10.1097/MOU.0000000000001163 10.1097/MOU.0000000000001163 PMC10990026 38426237

[5] Lee JR, Bang H, Dadhania D, Hartono C, Aull MJ, Satlin M et al Independent risk factors for urinary tract infection and for subsequent bacteremia or acute cellular rejection: A single-center report of 1166 kidney allograft recipients. Transplantation 2013 27;96:732–738. 10.1097/TP.0b013e3182a04997 10.1097/TP.0b013e3182a04997 PMC3833249 23917724

[6] Papasotiriou M, Savvidaki E, Kalliakmani P, Papachristou E, Marangos M, Fokaefs E et al (2011) Predisposing factors to the development of urinary tract infections in renal transplant recipients and the impact on the long-term graft function. Ren Fail 33:405–410. 10.3109/0886022X.2011.568137 21529269 10.3109/0886022X.2011.568137

[7] Evans L, Rhodes A, Alhazzani W, Massimo Antonelli CMC (2021) Surviving sepsis campaign: international guidelines for management of severe sepsis and septic shock 2021. 10.1007/s00134-008-1090-z

[8] Bonkat G (Chair), Bartoletti R, Bruyère TC F, Geerlings SE, Köves B, Schubert S, F. Wagenlehner Guidelines Associates: T. Mezei A, Pilatz B, Pradere RV (2020) EAU Guidelines on Urological Infections.;doi: https://uroweb.org/wp-content/uploads/EAU-Guidelines-on-Urological-infections-2020.pdf

[9] Goldman JD, Julian K (2019) Urinary tract infections in solid organ transplant recipients: guidelines from the American society of transplantation infectious diseases community of practice. Clin Transpl 133. 10.1111/ctr.13507 10.1111/ctr.13507 30793386

[10] Wagenlehner FME, Bjerklund Johansen TE, Cai T, Koves B, Kranz J, Pilatz A et al Epidemiology, definition and treatment of complicated urinary tract infections. Nat Rev Urol 2020 1;17:586–600. 10.1038/s41585-020-0362-4 10.1038/s41585-020-0362-4 32843751

[11] Hollyer I, Ison MG (2018) The challenge of urinary tract infections in renal transplant recipients. Transpl Infect Disease 20(1). 10.1111/tid.12828 10.1111/tid.12828 29272071

[12] Green H, Rahamimov R, Gafter U, Leibovitci L, Paul M (2011) Antibiotic prophylaxis for urinary tract infections in renal transplant recipients: A systematic review and meta-analysis. Transpl Infect Disease 13:441–447. 10.1111/j.1399-3062.2011.00644.x 21521435 10.1111/j.1399-3062.2011.00644.x

[13] Królicki T, Bardowska K, Kudla T, Królicka A, Letachowicz K, Mazanowska O et al Acute kidney injury secondary to urinary tract infection in kidney transplant recipients. Sci Rep [ Internet] 2022 1 [cited 2024 9];12. doi: https://pubmed.ncbi.nlm.nih.gov/35760823/doi: 10.1038/S41598-022-15035-7 10.1038/s41598-022-15035-7 PMC9237017 35760823

[14] Magiorakos AP, Srinivasan A, Carey RB, Carmeli Y, Falagas ME, Giske CG et al (2012) Multidrug-resistant, extensively drug-resistant and pandrug-resistant bacteria: an international expert proposal for interim standard definitions for acquired resistance. Clin Microbiol Infect 18:268–281. 10.1111/j.1469-0691.2011.03570.x 21793988 10.1111/j.1469-0691.2011.03570.x

[15] eucast: S I and R definitions [Internet]. [cited 2024 29];doi: https://www.eucast.org/newsiandr

[16] Humphries RM, Ambler J, Mitchell SL, Castanheira M, Dingle T, Hindler JA et al CLSI methods development and standardization working group best practices for evaluation of antimicrobial susceptibility tests. J Clin Microbiol [Internet] 2018 1 [cited 2024 9];56. doi: https://pubmed.ncbi.nlm.nih.gov/29367292/doi: 10.1128/JCM.01934-17 10.1128/JCM.01934-17 PMC5869819 29367292

[17] Analysis and Presentation of Cumulative Antimicrobial Susceptibility Test Data; Approved Guideline-Fourth Edition A guideline for global application developed through the Clinical and Laboratory Standards Institute consensus process (2014) [cited 2024 9];doi: www.clsi.org

[18] Fleischmann-Struzek C, Mellhammar L, Rose N, Cassini A, Rudd KE, Schlattmann P et al Incidence and mortality of hospital- and ICU-treated sepsis: results from an updated and expanded systematic review and meta-analysis. Intensive Care Med 2020 1;46:1552–1562. 10.1007/s00134-020-06151-x 10.1007/s00134-020-06151-x PMC7381468 32572531

[19] Tandogdu Z, Koves B, Ristovski S, Balci MBC, Rennesund K, Gravas S et al (2024) Urosepsis 30-day mortality, morbidity, and their risk factors: SERPENS study, a prospective, observational multi-center study. World J Urol 142. 10.1007/s00345-024-04979-2 10.1007/s00345-024-04979-2 PMC11087335 38730089

[20] Sawyer RG, Claridge JA, Nathens AB, Rotstein OD, Duane TM, Evans HL et al (2015) Trial of Short-Course antimicrobial therapy for intraabdominal infection. N Engl J Med 372:1996–2005. 10.1056/nejmoa1411162 25992746 10.1056/NEJMoa1411162 PMC4469182

[21] Yahav D, Franceschini E, Koppel F, Turjeman A, Babich T, Bitterman R et al Seven versus 14 days of antibiotic therapy for uncomplicated Gram-negative bacteremia: A noninferiority randomized controlled trial. Clin Infect Dis 2019 13;69:1091–1098. 10.1093/cid/ciy1054 10.1093/cid/ciy1054 30535100

[22] Kranz J, Bartoletti R, Bruyère F, Cai T, Geerlings S, Köves B et al European association of urology guidelines on urological infections: summary of the 2024 guidelines. Eur Urol 2024 1;86:27–41. 10.1016/j.eururo.2024.03.035 10.1016/j.eururo.2024.03.035 38714379

[23] Gupta K, Hooton TM, Naber KG, Wullt B, Colgan R, Miller LG et al (2011) 1;52 International clinical practice guidelines for the treatment of acute uncomplicated cystitis and pyelonephritis in women: A 2010 update by the Infectious Diseases Society of America and the European Society for Microbiology and Infectious Diseases. Clinical Infectious Diseases 10.1093/cid/ciq257 10.1093/cid/ciq257 21292654

[24] Holmbom M, Andersson M, Grabe M, Peeker R, Saudi A, Styrke J et al (2022) Community-onset urosepsis: incidence and risk factors for 30-day mortality–a retrospective cohort study. Scand J Urol 56:414–420. 10.1080/21681805.2022.2123039 36127849 10.1080/21681805.2022.2123039

[25] Ray AS, Haikal A, Hammoud KA, Yu ASL (2016) Vancomycin and the risk of AKI: A systematic review and meta-analysis. Clin J Am Soc Nephrol 11:2132–2140. 10.2215/CJN.05920616 27895134 10.2215/CJN.05920616 PMC5142072

[26] Bellos I, Karageorgiou V, Pergialiotis V, Perrea DN Acute kidney injury following the concurrent administration of antipseudomonal β-lactams and vancomycin: a network meta-analysis. Clin Microbiol Infect 2020 1;26:696–705. 10.1016/j.cmi.2020.03.019 10.1016/j.cmi.2020.03.019 32222460

[27] Luther MK, Timbrook TT, Caffrey AR, Dosa D, Lodise TP, Laplante KL Vancomycin plus Piperacillin-Tazobactam and acute kidney injury in adults: A systematic review and Meta-Analysis. Crit Care Med 2018 1;46:12–20. 10.1097/CCM.0000000000002769 10.1097/CCM.0000000000002769 29088001

[28] Martin T, Wilber E, Advani S, Torrisi J, Patel M, Rebolledo PA et al (2024) The impact of implementation of rapid blood culture identification panels on antimicrobial optimization: a retrospective cohort study. Antimicrob Stewardship Healthc Epidemiol 164. 10.1017/ash.2024.51 10.1017/ash.2024.51 PMC11019579 38628375

[29] Peri AM, Chatfield MD, Ling W, Furuya-Kanamori L, Harris PNA, Paterson DL Rapid diagnostic tests and antimicrobial stewardship programs for the management of bloodstream infection: what is their relative contribution to improving clinical outcomes?? A systematic review and network Meta-analysis. Clin Infect Dis 2024 15;79:502–515. 10.1093/cid/ciae234 10.1093/cid/ciae234 PMC11327801 38676943

