# Supplementary material for: Antibiotic practices in kidney transplant recipients with urosepsis are associated with treatment outcomes – a post-hoc analysis of an observational study
Source: Eur J Clin Microbiol Infect Dis. 2025 Jun 9;44(9):2129–38. doi: 10.1007/s10096-025-05181-x (PMC12457448; doi:10.1007/s10096-025-05181-x)
Supplement: Supplementary file 1 — Supplementary Material 1 [file 10096_2025_5181_MOESM1_ESM.docx]

Supplementary information

Antibiotic practices in kidney transplant recipients with urosepsis are associated with treatment outcomes – a post-hoc analysis of an observational study

Tomasz Królicki^1*^, Tobiasz Kudla^2^, Anna Królicka^1^, Klaudia Bardowska^3^, Krzysztof Letachowicz^4^, Ryszard Gawda^1^, Tomasz Czarnik^1^, Magdalena Krajewska^4^, Dorota Kamińska^4^

^1^ Department of Anesthesiology and Intensive Care, Institute of Medical Sciences, University of Opole, Poland

^2^ Department of Cardiology, American Heart of Poland, Kędzierzyn-Koźle, Poland

^3^ Department of Dermatology, Opole Voivodeship Hospital, Opole, Poland

^4^ Department of Non-Procedural Clinical Sciences, Faculty of Medicine, Wroclaw University of Science and Technology, Wroclaw, Poland

*corresponding author: Tomasz Królicki, t.krolicki105@gmail.com

Section 1

S1.1. Definitions and criteria utilized in the study

1. Urinary tract infection (UTI) – as defined by CDC/NHSM ^1^
2. Urosepsis (US) – UTI accompanied by proof of new organ dysfunction, defined as acute rise of SOFA score of ≥2 points ^2,3^.
3. Relapse of UTI – UTI within 14 days from antibiotic cessation for UTI or US, with the same bacterial isolate ^2^.
4. AKI (acute kidney injury) was defined as per KDIGO criteria ^4^.
5. Recovery from AKI was defined as difference between creatinine measured 1 month after discharge and the last control creatinine level before discharge lower or equal than ≥0.3mg/dL.
6. Recurrent urinary tract infection – was defined as per EAU definition as 2 UTI’s within 6 months or 3 episodes of UTI within 12 months ^2^.

S1.2. Microbiologic map:

To maximize isolates sample sizes, the microbiological map was created using a time span of 5 years (01.01.2024 – 31.12.2019) to maximize reliability of presented antibiotic resistance rates. Only microorganisms that were deemed causative for the US or UTI (strains that were identified as colonization or contamination were not reported) and the susceptibility profiles for the first isolate were reported if the same bacterial species were isolated over time. The created map does not duplicate bacteria isolated from different specimens (for example from blood or urine culture). If multiple isolated of the same bacteria species were obtained from specimens taken at the moment of admission, and discrepancies in susceptibility profiles existed, more resistant isolate was reported. The created map was created and presented with concordance with CLSI guidelines on Analysis and Presentation of Cumulative Antimicrobial Susceptibility Test Data ^5^.

Section 2 – Supplementary Tables

Supplementary Table S1

Table S1 – The prevalence of urosepsis, sepsis, urinary tract infection and all registered infections** in KTRs hospitalized in the Transplantation Unit during the study period (years 2014 - 2019).

| Year | **2014** | **2015** | **2016** | **2017** | **2018** | **2019** | **Sum from years 2014-2019** |
| --- | --- | --- | --- | --- | --- | --- | --- |
| Urosepsis  [n, % of all hospitalizations] | 15  (1.5%) | 21  (1.9%) | 20  (1.5%) | 35  (2.5%) | 31  (2.4%) | 17  (1.5%) | 139  (1.9%) |
| Sepsis* [n, % of all hospitalizations] | 17  (1.7%) | 22  (2%) | 30  (2.2%) | 33  (2.4%) | 35  (2.8%) | 26  (2.2%) | 163  (2.25%) |
| UTI [n, % of all hospitalizations] | 108  (11.1%) | 132  (12.1%) | 118  (8.8%) | 116  (8.3%) | 114  (9%) | 127  (10.9%) | 715  (9.87%) |
| All infections  [n, % of all hospitalizations] | 152  (15.6%) | 208  (19.1%) | 199  (14.8%) | 185  (13.2%) | 158  (12.5%) | 170  (14.5%) | 1072  (14.8%) |
| All hospitalizations of KTRs in Transplantation Clinic [n] | 973 | 1088 | 1343 | 1401 | 1269 | 1170 | 7244 |

*regardless of infection source, including US.
**bacterial, viral and fungal infections.

Supplementary Table S2

Table S2 - Epidemiological estimates of incidence of urosepsis, sepsis and urinary tract infection among the local population of kidney transplant recipients remaining under the care of the local Transplantation Unit.

|  | **Mean count per year:**  **[n / year]  (95% CI)** | **Estimated yearly incidence**  **[n / 1000 KTRs/ year]**  **(95% CI)** | **Estimated percentage of hospitalizations: [mean, 95% CI**]** |
| --- | --- | --- | --- |
| **Urosepsis** | 23.2  (16.8 – 29.6) | 21.1  (15.3 – 26.9) | 1.89%  (1.509% - 2.276%) |
| **Sepsis*** | 27.2  (21.7 – 32.6) | 24.7  (19.7 – 29.6) | 2.22%  (1.953% - 2.493%) |
| **UTI** | 119  (112 - 126) | 108  (102 -115) | 10.02%  (8.79% - 11.26%) |
| **All bacterial infections** | 179  (161 - 197) | 162  (146 - 179) | 14.96%  (13.09% - 16.83%) |

* regardless of infection source, including US.

**95% confidence intervals (CI) estimates for mean prevalence of each of the categories in relations to total hospitalization count.

Supplementary Table S3

Table S3 – Number of infections registered among hospitalized patients by source per study year.

| Type of infection by source: | 2014 | 2015 | 2016 | 2017 | 2018 | 2019 | Sum  (proportion of all hospitalizations) | Estimated percentage of all hospitalizations  [mean, 95% CI] |
| --- | --- | --- | --- | --- | --- | --- | --- | --- |
| UTI [n, %] | 108  (71%) | 132  (63,5%) | 118  (59,3%) | 116  (62,7%) | 114  (72,2%) | 127  (74,7%) | 715  (66,7%) | 67,2%  95% CI: 62,3% - 72,2% |
| Lower respiratory tract [n, %] | 23  (15,1%) | 37  (24,3%) | 32  (16,1%) | 24  (13%) | 18  (11,4%) | 17  (10%) | 151  (14,1%) | 13,9%  95% CI: 11,5% - 16,3% |
| Upper respiratory tract [n, %] | 8  (5,3%) | 17  (8,2%) | 25  (12,6%) | 29  (15,7%) | 13  (8,2%) | 11  (6,5%) | 103  (9,6%) | 9,4%  95% CI: 6,2% - 12,6% |
| Skin and soft tissue infection [n, %] | 5  (3,3%) | 7  (3,4%) | 12  (6%) | 10  (5,4%) | 0  (0%) | 3  (1,8%) | 37  (3,5%) | 3,5%  95% CI:1,5% - 5,1% |
| Infectious endocarditis [n, %] | 1  (0,7%) | 0  (0%) | 3  (3%) | 0  (0%) | 5  (3,2%) | 1  (0,6%) | 10  (0,9%) | 1%  95% CI: 0,02% - 2% |
| *Clostridioides difficile* [n, %] | 3  (2%) | 5  (2,4%) | 3  (3%) | 0  (0%) | 2  (1,3%) | 6  (3,5%) | 19  (1,8%) | 1,8%  95% CI: 0,8% - 2,7% |
| Acute intraabdominal infection or intraabdominal abscess [n, %] | 2  (1,3%) | 9  (4,3%) | 1  (0,5%) | 2  (1,1%) | 4  (2,5%) | 2  (1,2%) | 20  (1,9%) | 1,8%  95% CI: 0,7% - 2,9% |
| Other [n, %] | 2  (1,3%) | 1  (0,5%) | 5  (2,5%) | 4  (2,2%) | 2  (1,3%) | 3  (1,8%) | 17  (1,6%) | 1,6%  95% CI: 1% - 2,2% |
| Total: | 152 | 208 | 199 | 185 | 158 | 170 | 1072 |  |

Supplementary Table S4

Table S4 – Baseline characteristics of the study groups.

|  | **Urosepsis**  **(n=101)** | **UTI**  **(n=100)** | **p-value** |
| --- | --- | --- | --- |
| Age [years] (median, IQR) | 58 (44 - 66) | 56 (44 - 66) | 0.0193 |
| Sex [males / females] | 47 / 54 | 42 / 58 | 0.3068 |
| BMI [kg/m^2] (median, IQR) | 25.6 (21.4 – 29.7) | 24.6 (22.5 – 27.9) | 0.0555 |
| Length of stay [days] (median, IQR) | 14 (10 - 20) | 8 (6 - 13) | <0.0001 |
| CCI (Charlson-Comorbidity-Index) (median, IQR) | 5 (3 - 6) | 4 (3 - 5) | 0.0600 |
| History of recurrent UTI before inclusion [n, %] | 29 (28.7%) | 9 (9%) | 0.0002 |
| Time from transplantation to infection [months] (median, IQR) | 39.5 (4.7 - 130) | 49.1 (13.1 - 140) | 0.5254 |
| Baseline creatinine concentration [mg/dL]  (mean ± SD) | 1.56 ± 0.57 | 1.43 ± 0.43 | 0.1271 |
| Baseline eGFR [ml/min/1,73m^2^] (mean ± SD) | 45.5 ± 17.59 | 48.3 ± 13.82 | 0.0647 |
| Congenital anomaly of the genitourinary tract as cause of ESRD [n, %] | 10 (9.9%) | 6 (6%) | 0.3069 |
| Induction treatment before kidney transplantation (Basiliximab or Thymoglobulin) [n, %] | 14 (13.9%) | 20 (20%) | 0.2467 |
| Tripple maintenance immunosuppressive therapy prior at the moment of inclusion [n, %] | 74 (80.4%) | 84 (84%) | 0.0636 |

*BMI – body mass index, eGFR – effective glomerular filtration rate, ESRD – end stage renal disease, IQR – interquartile range, SD – standard deviation, UTI – urinary tract infection.*

Supplementary Table S5

Table S5 – Outcomes in the study group

|  | Urosepsis (n=101) | UTI (n=100) | p-value |
| --- | --- | --- | --- |
| In-hospital mortality [n, %] | 8 (7.9%) | 0 (0%) | 0.0120 |
| UTI-AKI [n, %] | 76 (75.2%) | 41 (41%) | <0.0001 |
| Need for dialysis initiation due to UTI/US [n, %] | 17 (16.8%) | 1 (1%) | <0.0001 |
| UTI relapse after discharge [n, %] | 15 (16.13%) | 1 (1%) | <0.0001 |
| Lack of recovery from AKI at 1 month [n, %]* | 28 (36.8%) | 16 (39.02%) | 0.8434 |
| Need for readmission for UTI at 1 year [n, %]** | 48 (51.6%) | 22 (22%) | <0.0001 |
| Reoccurrence of urosepsis at 1 year [n, %]** | 36 (38.7%) | n/a | n/a |

AKI – *acute kidney injury*, UTI - *urinary tract infection*, UTI-AKI - *urinary tract infection related acute kidney injury*, US – *urinary sepsis.*

*presented as percentage of patients who developed AKI.

**censored for death at 1-year.

Supplementary Table S6

Table S6 – Multivariable proportional Cox hazards model with stepwise predictor elimination for predictors of readmission for urinary tract infection at 1 year post discharge.

|  | **Univariable**  **p-value** | **Multivariable**  **p-value** | **Hazard**  **ratio** | **95% CI** |
| --- | --- | --- | --- | --- |
| Urosepsis | <0.0001 | 0.0033 | 3.01 | 1.657 – 5.485 |
| Urine outflow obstruction* | 0.0602 | 0.0122 | 2.03 | 1.167 – 3.521 |
| MDR strain UTI | 0.0065 | 0.0105 | 1.97 | 1.171 – 3.308 |
| History of recurrent UTI | <0.0001 | 0.0271 | 1.80 | 1.069 – 3.045 |
| Congenital urogenital anomaly as primary cause of ESRD | 0.0092 | 0.1744 | - | - |
| Time from transplantation <6 months ** | 0.0357 | 0.9697 | - | - |
| Baseline eGFR [ml/min/1,73m^2^] | 0.0336 | 0.1637 | - | - |
| Induction treatment | 0.1721 | 0.4470 | - | - |
| Age [years] | 0.6092 | 0.8964 | - | - |
| Sex [males / females] | 0.8501 | 0.7773 |  |  |
| Prophylactic antibiotic therapy post discharge | 0.8929 | 0.8259 | - | - |
| Triple maintenance immunosuppressive therapy** | 0.9101 | 0.7626 | - | - |
| Charlson Comorbidity Index (CCI)** | 0.9369 | 0.5240 | - | - |

Adjusted R^2^ = 0,4851, 95% CI: 0,3783 - 0,5919, p<0,0001.

**Both lower and upper urinary tract obstruction, ** assessed at the moment of inclusion in the study.*

Variables with p<0.05 in the univariable analysis were included. Additionally the authors have decided to include variables that appeared as non-significant but are important in the clinical context.

Section 3

Assessment of Proportional Hazard assumption in the multivariable model presented in Tabel S6.

| **PH TEST** | | | | | |
| --- | --- | --- | --- | --- | --- |
| Variable_level |  | |  | |  |
|  | Chi-square | df | | p-value | |
| Urine outflow obstruction_0 | 2.100856 | 1 | | 0.147217 | |
| MDR infection_0 | 3.122589 | 1 | | 0.077214 | |
| urosepsis_0 | 0.022204 | 1 | | 0.881546 | |
| Prior_recurrent_UTI_0 | 0.013258 | 1 | | 0.908332 | |
| Overall | 5.737124 | 4 | | 0.219659 | |

p-value >0.05 indicates that the PH assumption is not violated.

Schoenfeld plots were generated for each variable.

Figure S1 – Schoenfeld residual scatterplots for MDR infection
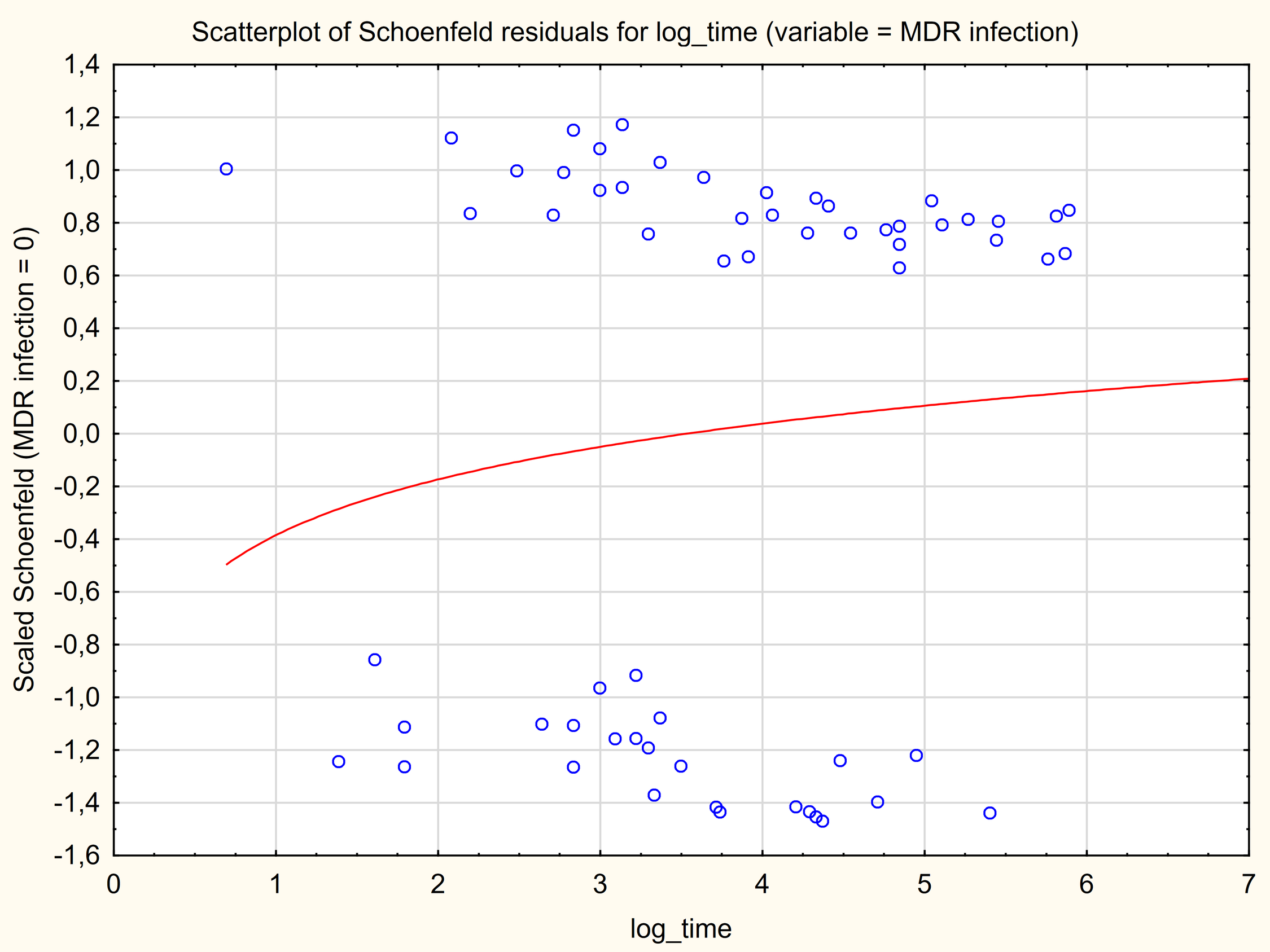


Figure S2 – Schoenfeld residual scatterplots for prior recurrent UTI


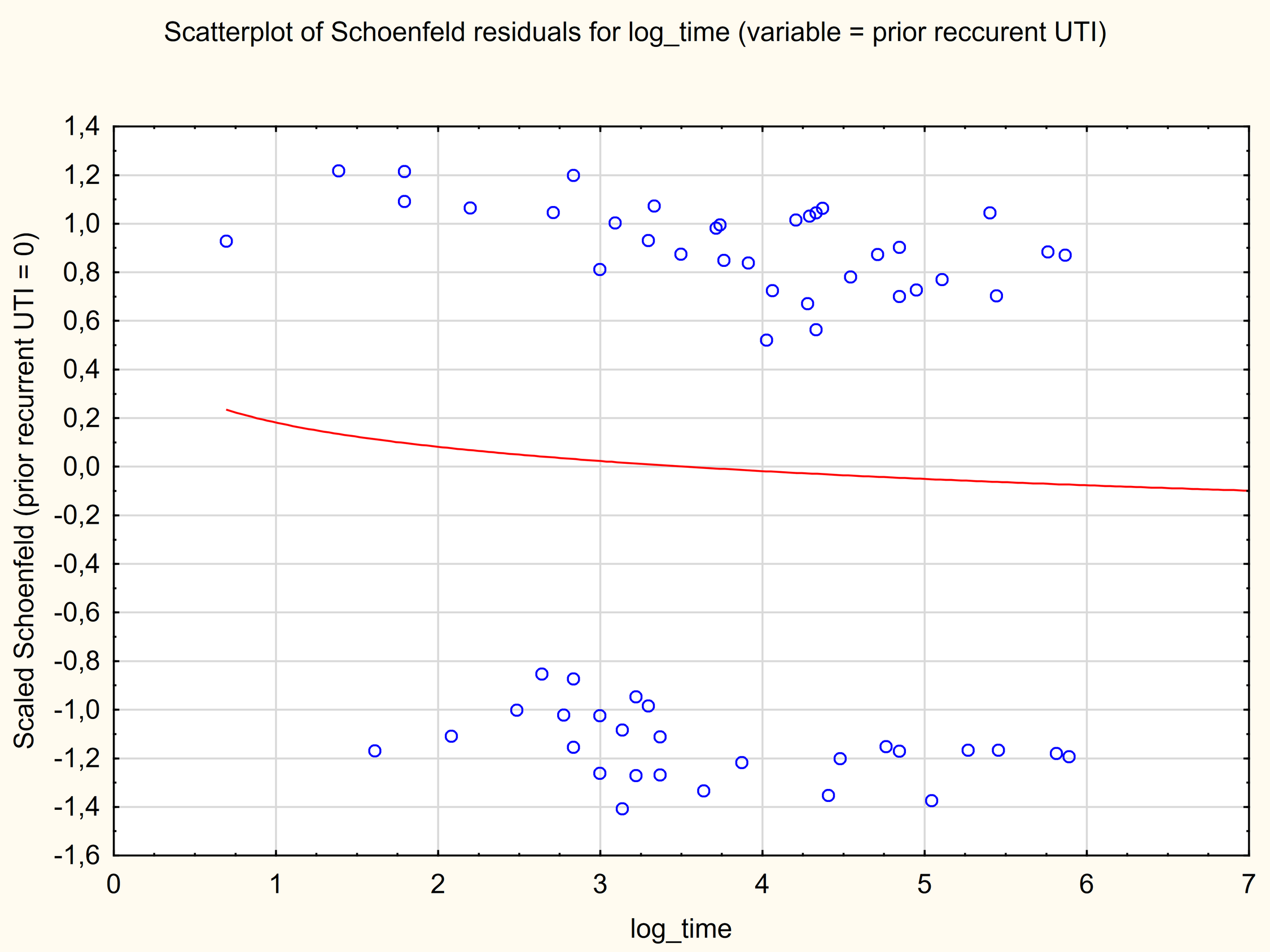


Figure S3 – Schoenfeld residual scatterplots for urine outflow obstruction at the moment of study inclusion


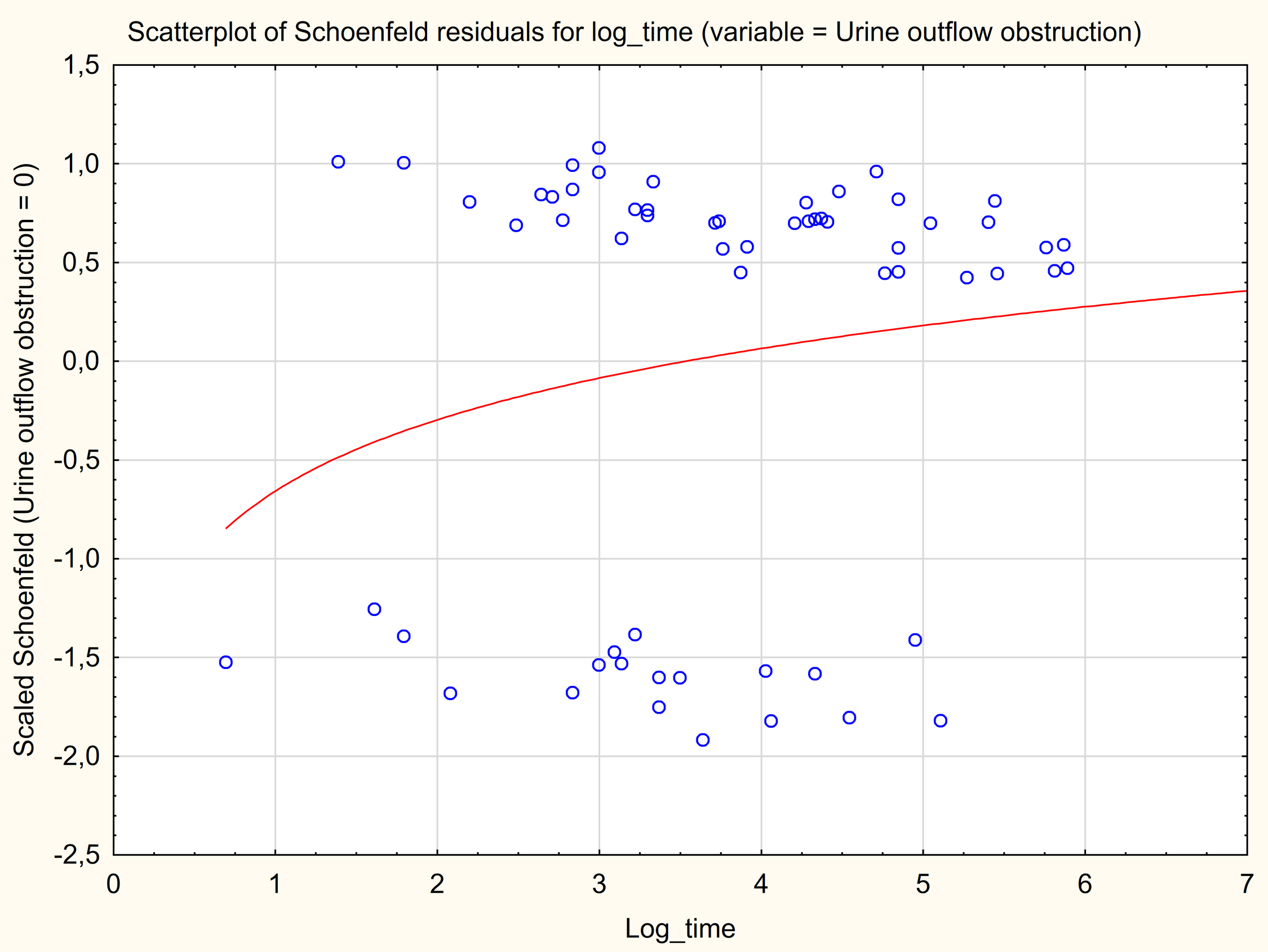


Figure S4 – Schoenfeld residual scatterplots for urosepsis


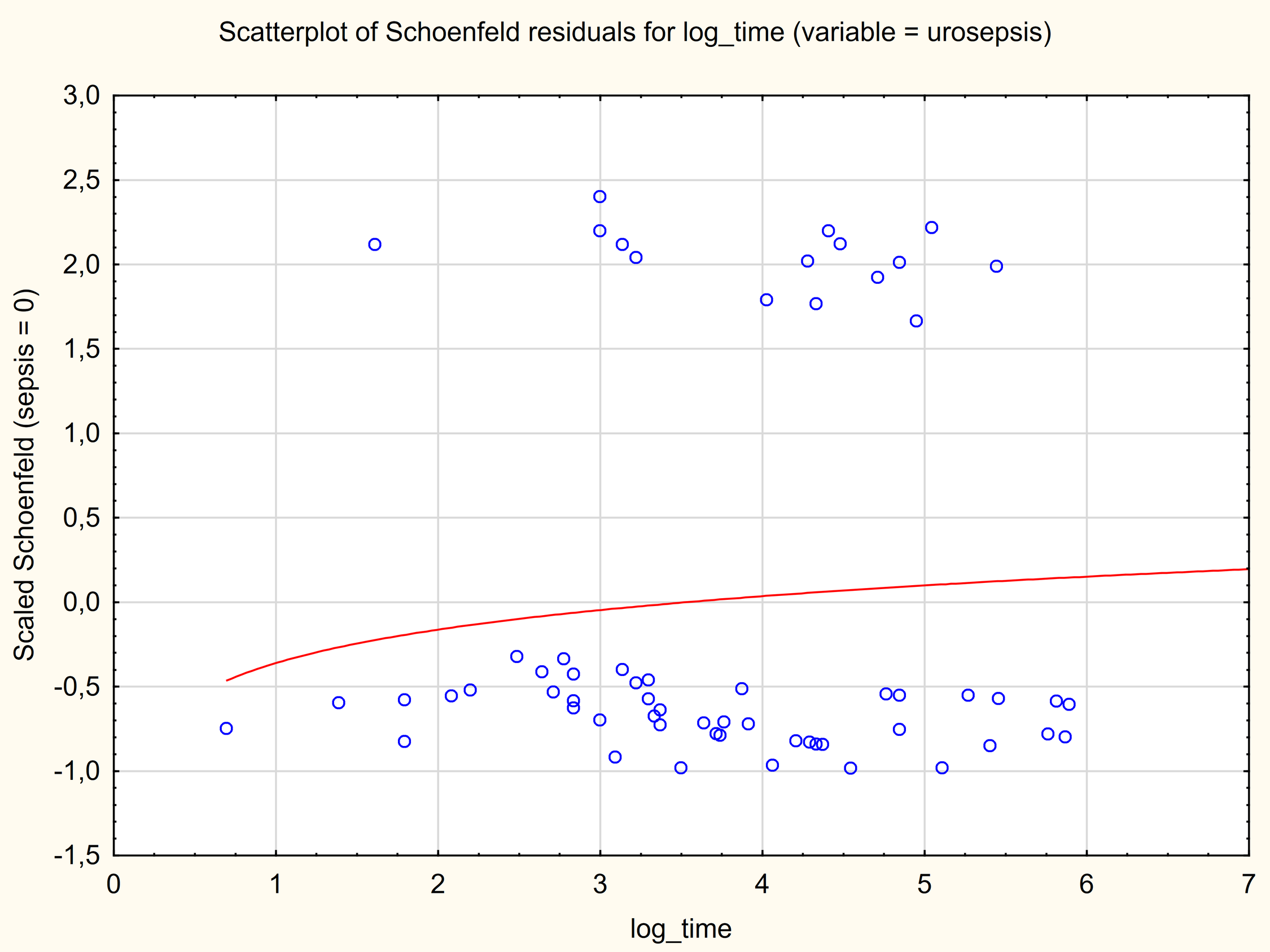


**References:**

1. Horan, T. C., Andrus, M. & Dudeck, M. A. CDC/NHSN surveillance definition of health care-associated infection and criteria for specific types of infections in the acute care setting. *Am J Infect Control* **36**, 309–332 (2008).

2. G. Bonkat (Chair), R. Bartoletti, F. Bruyère, T. C. & S.E. Geerlings, B. Köves, S. Schubert, F. Wagenlehner Guidelines Associates: T. Mezei, A. Pilatz, B. Pradere, R. V. EAU Guidelines on Urological Infections. (2020).

3. Laura Evans, Andrew Rhodes, Waleed Alhazzani, Massimo Antonelli, C. M. C. *Surviving Sepsis Campaign: International Guidelines for Management of Severe Sepsis and Septic Shock 2021*. *Intensive Care Medicine* vol. 34 (2021).

4. Kellum, J. A. *et al.* Kidney disease: Improving global outcomes (KDIGO) acute kidney injury work group. KDIGO clinical practice guideline for acute kidney injury. *Kidney International Supplements* vol. 2 1–138 Preprint at https://doi.org/10.1038/kisup.2012.1 (2012).

5. Analysis and Presentation of Cumulative Antimicrobial Susceptibility Test Data; Approved Guideline-Fourth Edition A guideline for global application developed through the Clinical and Laboratory Standards Institute consensus process. (2014).
